# Site- and Size-Based Algorithm for Reconstruction of Cheek Skin Defects: A Single-Center Retrospective Study

**DOI:** 10.3390/jcm15010331

**Published:** 2026-01-01

**Authors:** Emilia Lis, Anna Lato, Julia Miaśkiewicz, Michał Gontarz, Tomasz Marecik, Krzysztof Gąsiorowski, Grażyna Wyszyńska-Pawelec, Jakub Bargiel

**Affiliations:** 1 Students’ Scientific Group, Department of Cranio-Maxillofacial Surgery, Jagiellonian University Medical College, 31-501 Krakow, Poland; emilia.weronika.lis@gmail.com (E.L.); anna.lato@student.uj.edu.pl (A.L.); julia.miaskiewicz@student.uj.edu.pl (J.M.); 2 Department of Cranio-Maxillofacial Surgery, Jagiellonian University Medical College, 31-501 Krakow, Polandtomasz.marecik@uj.edu.pl (T.M.); krzysztof.gasiorowski@uj.edu.pl (K.G.); grazyna.wyszynska-pawelec@uj.edu.pl (G.W.-P.)

**Keywords:** non-melanoma skin cancer, basal cell carcinoma, squamous cell carcinoma, cheek, reconstructive surgical procedures, skin grafting, local flaps, treatment algorithm

## Abstract

**Background:** The rising incidence of cutaneous non-melanoma skin cancers underscores the need for individualized reconstruction, particularly for cheek defects that pose distinctive anatomic and functional challenges. This study aimed to analyze reconstructive patterns for cheek skin lesions and to develop a simple, site- and size-based algorithm for small- to medium-sized defects. **Methods**: We retrospectively reviewed 129 consecutive patients treated between 2022 and 2025 for primary basal cell carcinoma, squamous cell carcinoma, or benign cheek skin tumors. After excision, defects were reconstructed with primary closure, local flaps, or skin grafts. Associations between the largest clinically measured lesion diameter (used as a proxy for the post-excision defect size), anatomical subsite, histopathology, and reconstructive technique were evaluated using ANOVA or Kruskal–Wallis tests, chi-square tests, and Spearman’s correlation. **Results**: The mean lesion diameter was 19.75 ± 12.93 mm. Reconstruction was performed using local flaps in 62 patients (48.06%), primary closure in 53 (41.09%), and skin grafts in 14 (10.85%). Larger defects were more frequently managed with grafts or flaps (F(2,110) = 4.84, *p* = 0.010), and lesion size correlated with reconstructive complexity (Spearman’s ρ = 0.229, *p* = 0.015). Lesion location was also significantly associated with the reconstruction method (χ^2^(10) = 48.29, *p* < 0.001; Cramér’s V = 0.44). Margin-negative (R0) excision was achieved in 95.35% of cases, with a low recurrence rate (3.91%) and complication rate (1.56%). **Conclusions:** Lesion size and anatomical location are key determinants of reconstructive strategy for cheek skin defects. In this cohort, lesions ≤ 20 mm were predominantly managed with primary closure, whereas lesions > 20 mm more frequently required flap reconstruction or skin grafting. This size-based split is cohort-derived and should be interpreted as a pragmatic framework that requires external validation.

## 1. Introduction

The human face is a cornerstone of nonverbal communication, and any disruption of its structure can cause profound psychological distress and social impairment [1]. Reconstructive efforts aimed at restoring facial form and function have been documented since antiquity. As surgical techniques have evolved, the pursuit of optimal aesthetic outcomes has remained a primary objective. In recent years, the incidence of non-melanoma skin cancer (NMSC) affecting the head and neck has continued to increase, highlighting the ongoing need to refine reconstructive techniques [2]. The most common benign facial lesions include pigmented nevi and epidermal cysts [3,4]. Among malignant tumors, basal cell carcinoma (BCC) is the most prevalent, followed by squamous cell carcinoma (SCC). Nevertheless, some studies suggest that SCC may be more common than BCC among cutaneous cheek lesions [5,6].

Anatomically, the cheek is composed of skin, subcutaneous fat (including the buccal fat pad), muscles, submucosa, and mucosa. The overlying skin is thin, highly vascularized, and contains both sebaceous glands and hair follicles. Deep to the skin lies the buccinator muscle, which compresses the cheek against the teeth during mastication and prevents food from accumulating in the oral vestibule. Additional muscles, including the zygomaticus major and risorius, contribute to facial expression and mobility. Sensory innervation is primarily provided by the buccal branch of the mandibular nerve, whereas the buccal branches of the facial nerve supply motor innervation to the buccinator and other facial expression muscles in this area. The vascular supply primarily originates from branches of the facial artery, particularly the buccal and inferior labial arteries, as well as the transverse facial artery, a branch of the superficial temporal artery. Venous drainage closely parallels the arterial system and occurs primarily via the facial vein. Functionally, the cheek plays a vital role in mastication, speech, facial expression, and sensory perception, while also contributing to the structural contour and protection of the face [7].

Cheek reconstruction presents a considerable challenge due to the region’s anatomical complexity and heterogeneous tissue characteristics, including variable skin thickness, elasticity, and contour. These factors, coupled with the functional and aesthetic importance of the cheek area, necessitate a patient-specific, defect-oriented reconstructive strategy. Effective management requires a comprehensive assessment of the defect’s dimensions, depth, and precise topographical location within the cheek subunits, accounting for both anatomical intricacies and aesthetic considerations. A broad spectrum of reconstructive options is available, ranging from primary closure and skin grafting to local, regional, and microsurgical free flaps. The chosen technique should strike a balance between functional restoration and aesthetic outcome. Key considerations include preserving perioral and periorbital mobility, adhering to aesthetic subunit principles, and maintaining natural facial landmarks, such as skin creases and contour lines.

For minor facial defects, typically less than 2–3 cm in diameter, excision parallel to relaxed skin tension lines (RSTL) with primary closure is generally preferred owing to its simplicity, low morbidity, and excellent color and texture match with the adjacent skin. This approach is practical in elderly patients, in whom increased skin laxity allows for tension-free closure [8]. Medium-sized or more complex defects often require the use of local flaps—such as advancement, rotation, or nasolabial flaps—which transfer adjacent tissue with similar characteristics while maintaining key facial landmarks [9,10]. When primary closure or local flaps are not feasible, skin grafting provides a practical alternative. Full-thickness grafts are typically preferred over split-thickness variants due to their superior aesthetic integration, particularly in terms of color and texture match. Although skin grafts may not fully restore three-dimensional contour, they provide dependable reconstruction with minimal donor-site morbidity [11]. In cases where local tissue is inadequate—due to extensive defects, prior scarring, or poor skin quality—regional flaps, such as the cervicofacial flap, become viable options, especially for defects exceeding 4–5 cm in diameter [12]. For extensive or full-thickness defects involving deeper anatomical layers, such as bone or intraoral structures, microvascular free tissue transfer is often required. Free flaps, such as the radial forearm and anterolateral thigh flaps, provide well-vascularized tissue with adjustable volume, enabling reliable reconstruction even in previously irradiated or surgically altered fields [13]. Although functional and aesthetic restoration after excision of cheek skin neoplasms is essential, the primary objective remains complete oncologic resection with adequate margins. Because these tumors are typically diagnosed at an early stage, defects at the time of excision rarely exceed 20 mm and are usually suitable for primary closure or local flap reconstruction [14].

Therefore, there is a need for simple, data-driven decision tools that guide the choice between primary closure, local flaps, and skin grafts for cheek skin defects, particularly in the setting of non-melanoma skin cancer. The present study aimed to characterize reconstructive patterns for cheek skin lesions in a consecutive single-center cohort and develop a site- and size-stratified algorithm for selecting the optimal reconstruction method for small- to medium-sized defects while maintaining oncologic safety.

## 2. Materials and Methods

This retrospective observational cohort study included 129 patients who underwent surgical treatment for cheek skin lesions between January 2022 and March 2025 at the Department of Cranio-Maxillofacial Surgery, Jagiellonian University Hospital in Krakow, Poland. Eligible patients presented for the first time with a cheek skin lesion that was subsequently confirmed histopathologically as non-melanoma skin cancer or a benign tumor. Lesions measuring ≤ 1 cm in greatest diameter were managed by complete excision (excisional biopsy), whereas larger lesions underwent incisional biopsy before definitive surgical intervention. Patients were excluded if the lesion extended to the nasal or periorbital region (excluding the medial canthus and lower eyelid), involved the buccal mucosa, or represented recurrent disease. The study was conducted in accordance with the Declaration of Helsinki and approved by the Bioethics Committee of the Jagiellonian University (protocol code 1072.6120.230.2021, approved on 29 September 2021).

The choice of reconstructive technique was determined by a multidisciplinary skin tumor board, considering factors such as patient age, overall health status, histopathological findings, and tumor stage. Local flaps and primary closure were the most frequently employed methods, reflecting the study’s focus on small- to medium-sized defects. In patients with significant comorbidities, reconstruction techniques amenable to local anesthesia were preferred to reduce perioperative risk.

Statistical analyses were conducted to assess patient demographics (age, gender, weight, height, body mass index [BMI], history of alcohol use and smoking), excised lesion characteristics (type, location, laterality, and size), surgical procedure and hospitalization duration, type of anesthesia, reconstruction method, postoperative complications, and follow-up visits.

Lesion size was defined as the greatest clinical diameter of the cutaneous lesion recorded in millimeters. For malignant tumors, excision margins were planned according to institutional guidelines (4 mm for basal cell carcinoma and 6 mm for squamous cell carcinoma). From a reconstructive standpoint, this 2 mm per-side difference is unlikely to influence technique selection in the present cohort. Accordingly, the expected post-excision defect diameter is typically larger than the recorded lesion diameter by approximately 8–12 mm, depending on diagnosis and margin design. Because post-excision defect dimensions, depth, and closure tension were not documented in a standardized manner in this retrospective dataset, lesion diameter was used as a pragmatic, consistently available surrogate to maintain transparency and reproducibility of the protocol.

Statistical analyses were conducted using IBM SPSS Statistics, version 29.0.2.0 (IBM Corp., Armonk, NY, USA). The Shapiro–Wilk test was used to assess normality. Depending on distribution and homogeneity of variance (Levene’s test), group comparisons for continuous variables were performed using one-way ANOVA or the Kruskal–Wallis test. When overall tests were significant, pairwise comparisons were conducted using the Games–Howell test (for ANOVA with unequal variances) or the Mann–Whitney U test, as appropriate. Categorical variables were analyzed with the chi-square test of independence, and effect sizes were expressed as Cramér’s V. Spearman’s rank correlation was used to assess associations between lesion size and reconstructive complexity. A *p*-value < 0.05 was considered statistically significant. Multiple pairwise tests were adjusted for Bonferroni.

**Figure 1:**
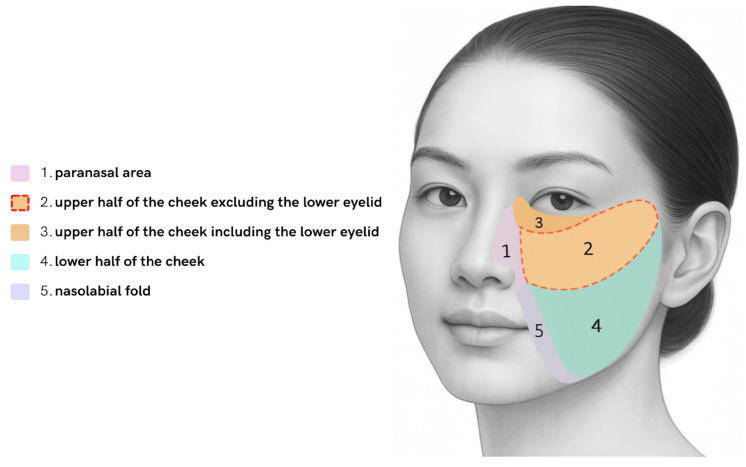
Anatomical categorization of the cheek area into five regions: (1) paranasal area, (2) upper half excluding the lower eyelid, (3) upper half including the lower eyelid, (4) lower half, and (5) nasolabial fold. Schematic created by the authors with the assistance of AI.

To accurately illustrate lesion distribution and the corresponding surgical approaches, the cheek region was divided into five anatomical regions: (1) the paranasal area, (2) the upper half excluding the lower eyelid, (3) the upper half including the lower eyelid, (4) the lower half, and (5) the nasolabial fold (Figure 1).

## 3. Results

### 3.1. Study Group Characteristics

A total of 129 patients with cheek skin lesions treated between 2022 and 2025 were included in the analysis. The cohort comprised 60 men (46.51%) and 69 women (53.49%), yielding a male-to-female ratio of 0.87:1. The mean age was 64.51 ± 18.56 years, and the mean body mass index (BMI) was 26.54 ± 4.70 kg/m^2^. Thirty patients (23.26%) reported a history of smoking. Regarding alcohol consumption, 28 individuals (21.70%) were classified as occasional drinkers (alcohol intake fewer than three times per week), whereas the remaining 101 (78.30%) reported abstaining.

**Figure 2:**
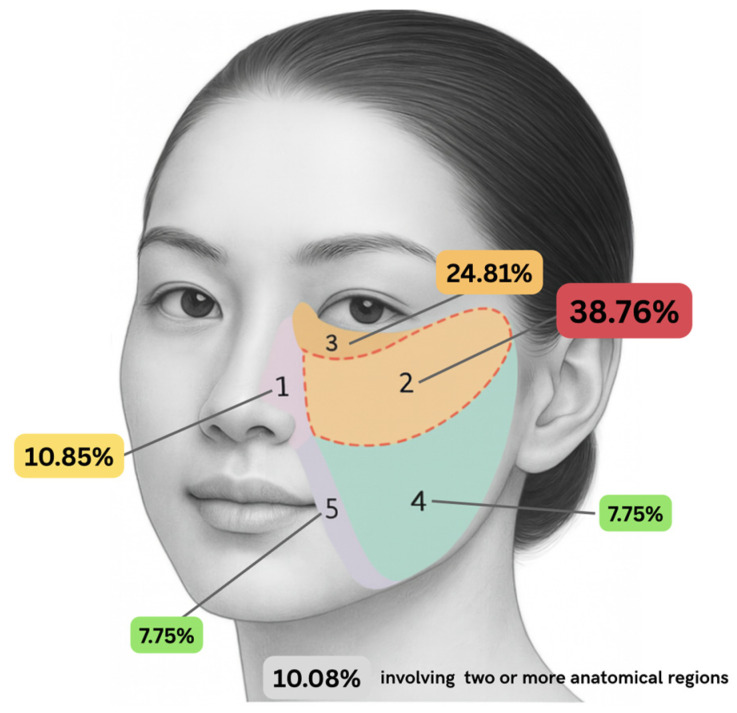
Schematic distribution of the frequency of skin lesions in selected cheek regions (%). Schematic created by the authors with the assistance of AI.

The most frequent diagnosis in this cohort was BCC, identified in 61 patients (47.29%), followed by benign lesions in 46 patients (35.66%) and SCC in 22 patients (17.05%). Tumors were most commonly located on the right side of the face (86 cases, 66.67%). The most frequent lesion site was region 2, observed in 50 cases (38.76%), followed by region 3 with 32 cases (24.81%). Lesions occurred in region 1 in 14 patients (10.85%), region 4 in 10 patients (7.75%), and region 5 in another 10 patients (7.75%). In 13 cases (10.08%), lesions involved two or more areas (Figure 2).

Lesion size ranged from 1.4 to 65 mm, with a mean of 19.75 ± 12.93 mm. Most lesions measured ≤ 20 mm in diameter (51.33%), 42.28% measured 21–40 mm, and only 6.39% exceeded 40 mm. Skin defects were most frequently reconstructed with local flaps (62 patients, 48.06%), followed by primary closure (53 patients, 41.09%) and skin grafts (14 patients, 10.85%). Baseline demographic, clinical, and histopathological characteristics of the study cohort are summarized in Table 1.

The mean surgical time was 63.32 ± 36.05 min, and the mean hospital stay was 2.59 ± 3.56 days. Local anesthesia was utilized in 96 procedures (74.42%), while general anesthesia was required in 33 cases (25.58%). The choice of anesthesia was associated with lesion diameter and the planned reconstructive method (*p* = 0.047). Procedures performed under local anesthesia involved smaller lesions (mean diameter 18.75 mm) than those performed under general anesthesia (mean diameter 22.98 mm). Primary closure and local flaps were predominantly performed under local anesthesia (94.34% and 66.13%, respectively), whereas skin grafting was more frequently performed under general anesthesia (64.29%; *p* < 0.001).

All excised specimens underwent histopathological evaluation. Incomplete excision was defined as the presence of tumor cells ≤ 1 mm from the lateral or deep surgical margins. According to the institutional guidelines, a minimum surgical margin of 4 mm was targeted for BCC and 6 mm for SCC. Radical (R0) excision was achieved in 123 patients (95.35%), while six patients (4.65%) underwent non-radical (R1) resections, including four BCCs and two SCCs. In cases with R1 resection, re-excision was performed to achieve adequate surgical margins. All R1 resections occurred in patients reconstructed with local flaps. However, this factor was not included in the outcome analysis.

Local recurrence was observed in 5 cases (3.91%), of which four patients had initially undergone R0 resection. Surgical complications occurred in 2 patients (1.56%), both of whom underwent reconstruction with local flaps. One patient developed wound dehiscence with a minor hematoma, and the other experienced dehiscence with purulent discharge. Both cases were successfully managed with conservative treatment. Overall, aesthetic and functional outcomes were satisfactory in nearly all patients, as assessed clinically during scheduled follow-up visits. Scar revision for aesthetic improvement was performed in 5 individuals (3.91%)—two from the primary closure group and three from the local flap group. In contrast, the remaining patients exhibited no functional deficits and did not report aesthetic concerns.

The characteristics of surgical procedures and early oncologic outcomes are summarized in Table 2.

### 3.2. Stratified Analysis by Reconstructive Modality

Patient characteristics across surgical technique subgroups were generally comparable with respect to gender, BMI, smoking, and alcohol consumption (all *p* > 0.05). However, statistically significant differences were observed between groups in patient age (with skin graft recipients representing the oldest cohort) and lesion location. Among patients in the primary closure group, lesions were most frequently localized to the upper half of the cheek (23 cases, 43.40%). In contrast, involvement of more than two anatomical regions was the least common finding (2 cases, 3.77%). In the skin graft group, lesions most often encompassed more than two anatomical regions (8 cases, 57.14%) and the upper half of the cheek extending to the lower eyelid region (5 cases, 35.71%). In the local flap group, lesions were predominantly located in the upper half of the cheek (26 cases, 41.94%), whereas involvement of the lower cheek was rare (1 case, 1.61%).

Furthermore, lesion size differed significantly among subgroups (*p* = 0.03), with mean dimensions of 15.72 ± 9.22 mm for primary closure, 26.00 ± 19.38 mm for skin grafts, and 21.98 ± 13.21 mm for local flaps. The type of reconstruction was significantly associated with histopathologic category (*p* < 0.001). SCC was most common in the skin graft and local flap groups and was absent in the primary closure group. Benign lesions were most frequently observed in the primary closure group.

Demographic and clinical characteristics of patients according to surgical technique are summarized in Table 3.

Significant intergroup differences were observed in the surgical duration, length of hospitalization, resection type, and anesthesia method. Procedures involving skin grafts were the most time-consuming (mean 88.89 ± 51.28 min), whereas those utilizing local flaps were the shortest (mean 56.88 ± 29.22 min; *p* < 0.001). Hospital stay was significantly longer in the skin graft group (5.62 ± 2.63 days) compared with primary closure (1.23 ± 0.78 days) and local flaps (3.18 ± 4.66 days; *p* < 0.001). The type of anesthesia differed significantly between groups (*p* < 0.001): local anesthesia predominated in the primary closure group (94.34%), whereas general anesthesia was more frequently employed in the skin graft (64.29%) and local flap (33.87%) groups.

Complete (R0) excision was achieved in all primary closure and skin graft cases, whereas incomplete (R1) margins were observed in 9.84% of local flap cases (*p* = 0.03). No statistically significant differences were identified in local recurrence rates (*p* = 0.169), recurrence onset time (*p* = 0.616), or the need for secondary corrective procedures (*p* = 0.652). All patients in the primary closure group survived throughout the follow-up period, which lasted at least 6 months, while mortality was observed in 7.14% of the skin graft group and 9.68% of the local flap group (*p* = 0.05).

Perioperative and early oncologic outcomes according to surgical technique are summarized in Table 4.

#### 3.2.1. The Impact of Patients’ Age on the Selection of Surgical Technique

A Kruskal–Wallis test revealed a statistically significant difference in patient age across the three surgical techniques (H(2) = 10.43, *p* = 0.005). Post hoc pairwise comparisons using the Mann–Whitney U test with Bonferroni correction showed that patients undergoing skin grafts were significantly older than those undergoing primary closure (*p* < 0.05). There were no significant differences in age between the flap and primary closure group and between the flap and skin graft group. These results suggest that age was a contributing factor in selecting grafts over primary closure but did not significantly influence the choice between flaps and other techniques. Spearman’s rank correlation showed no significant monotonic relationship between patient age and surgical technique (r = 0.043, *p* = 0.629). Although age differed significantly between groups, it did not follow a consistent trend across surgical methods.

#### 3.2.2. The Association Between the Histopathological Diagnosis and Choice of Surgical Technique

A statistically significant association was identified between histopathological diagnosis and surgical technique (χ^2^, *p* < 0.001; Cramér’s V = 0.316). BCC was managed using all three reconstruction methods, most frequently with flap reconstruction. SCC was managed exclusively with grafts and flaps, with no cases managed by primary closure. Benign lesions were reconstructed using flaps or primary closure, but never with grafts. When comparing benign lesions with malignant lesions (BCC and SCC), no statistically significant difference in size was observed, with benign lesions averaging 18.63 mm and BCC and SCC lesions averaging 21.23 mm (*p* > 0.05). These findings indicate that the choice of reconstructive technique was influenced by lesion type, with BCCs and SCCs favoring grafts and flaps over primary closure.

#### 3.2.3. The Association Between the Lesion Size and Choice of Surgical Technique

A one-way analysis of variance (ANOVA) was performed to evaluate the relationship between reconstructive technique and the size of cheek skin defects. The study demonstrated a statistically significant effect of reconstruction method on lesion size, F(2,110) = 4.84, *p* = 0.010, with an effect size of η^2^ = 0.081, indicating that approximately 8.1% of the variance in lesion size could be attributed to the reconstruction method. Levene’s test revealed a significant violation of the assumption of homogeneity of variances (*p* < 0.05); therefore, the Games–Howell post hoc test was applied. Post hoc comparisons showed that lesions managed with primary closure (mean = 15.72 mm) were significantly smaller than those reconstructed using skin grafts (mean = 26.00 mm; *p* = 0.018) or local flaps (mean = 21.98 mm; *p* = 0.018), whereas no significant difference was observed between the graft and flap groups (*p* > 0.05). To further substantiate this relationship, Spearman’s rank correlation analysis demonstrated a statistically significant positive association between lesion size and reconstructive method (ρ = 0.229, *p* = 0.015), confirming that larger defects were more frequently managed with advanced reconstructive approaches, including skin grafts and local flaps, rather than primary closure. Mean lesion size and intergroup differences across surgical techniques are presented in Table 5.

**Figure 3:**
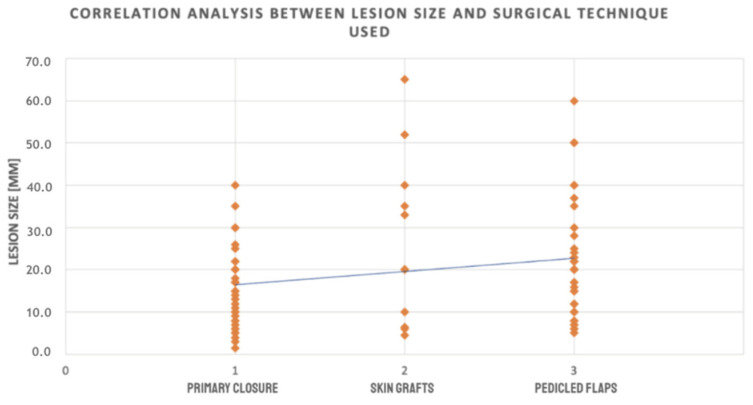
Scatter plot showing the distribution of lesion sizes by type of surgical procedure. Each point represents an individual case. A linear regression line is included to illustrate the trend, indicating a tendency toward the use of more complex reconstructive techniques for larger lesions.

Collectively, these findings provide consistent evidence that the extent of tissue loss significantly influences the choice of reconstructive technique, with primary closure favored for smaller lesions and local flaps or skin grafts increasingly required as defect size increases (Figure 3).

#### 3.2.4. The Association Between Lesion Location and the Choice of Surgical Technique

**Figure 4:**
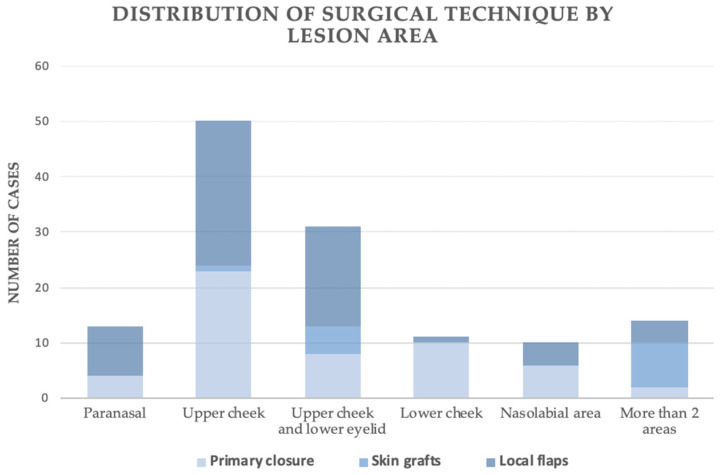
Distribution of surgical technique by lesion site.

Lesion location across five facial subsites was significantly associated with the reconstructive technique employed (χ^2^(10) = 48.29, *p* < 0.001). The strength of this association was moderate (Cramér’s V = 0.44). Within the primary closure group, lesions most frequently involved the upper or lower cheek, whereas this technique was rarely applied to paranasal tumors or multi-region defects. Skin grafts were predominantly used for multi-region defects and for upper-cheek lesions extending into the lower eyelid, with no grafts performed in the paranasal area. Local flaps were preferred for lesions of the upper cheek, periorbital region, and paranasal area, where their use exceeded that of the other two methods. Still, they were least employed for isolated lower-cheek lesions. Collectively, these findings indicate that the anatomical location of the defect plays a critical role in determining the reconstructive approach (Figure 4).

## 4. Discussion

Non-melanoma skin cancer is one of the most common malignancies worldwide, and basal cell carcinoma, together with squamous cell carcinoma, accounts for the vast majority of cases [5,15,16]. In line with previous epidemiological studies, basal cell carcinoma represented almost half of all lesions in our series and squamous cell carcinoma a smaller but clinically meaningful proportion [5,17,18,19]. Basal cell carcinoma typically exhibits slow local growth and low metastatic potential. In contrast, squamous cell carcinoma is more aggressive and carries a higher risk of nodal and distant spread, even though early-stage disease can still achieve excellent 5-year survival [16,18,19,20]. Because these tumors develop on visible and functionally essential parts of the face, such as the cheek, surgical treatment must combine complete oncologic clearance with careful planning of the reconstructive procedure.

The demographic profile of our patients is broadly comparable with that reported in earlier work. The mean age of about sixty-five years confirms that cheek skin tumors predominantly affect older adults [21,22]. In our cohort, women were slightly more numerous than men, in contrast to many studies that report a male predominance in facial NMSC [23,24]. This discrepancy may reflect local differences in health-seeking behavior, referral patterns, or patterns of ultraviolet exposure, but the design of this investigation does not allow firm conclusions. The mean BMI indicated that most patients were overweight. Previous extensive cohort studies have suggested a somewhat lower risk of non-melanoma skin cancer in overweight women than in women of normal weight [25,26]. In contrast, alcohol intake has been repeatedly linked to a dose-dependent increase in the risk of NMSC [27,28]. In our series, most patients reported no regular alcohol consumption, which again shows that a single-center surgical cohort is better suited to describe clinical and reconstructive aspects than to quantify lifestyle-related risk.

The anatomical distribution of lesions in the present study showed several patterns directly relevant to reconstruction. Tumors were more often located on the right cheek than on the left, which agrees with earlier reports of right-sided predominance for basal cell carcinoma, SCC, and benign facial tumors. This has been attributed to asymmetric ultraviolet exposure during driving and other daily activities [29]. Within the cheek, lesions most commonly involved the upper half, sometimes extending into the lower eyelid region, whereas the lower cheek was less frequently affected. Other authors have also highlighted the upper cheek, periorbital, and paranasal regions as common locations, while some have described a predominance of lesions in the nasolabial fold [30,31,32,33,34,35]. Our five-region subdivision of the cheek allowed us to show that multi-regional and periorbital paranasal defects are particularly challenging and often require more advanced reconstruction.

A broad spectrum of reconstructive options is available for cheek defects. These range from primary closure and skin grafting to local and regional flaps and finally to free tissue transfer [30,31,36,37,38,39]. In everyday clinical practice, most defects seen by dermatologic, maxillofacial, and plastic surgeons are small or medium in size. They can be treated with primary closure, local flaps, or skin grafts. Our cohort reflects this situation. Very large or full-thickness defects requiring regional or free flaps were uncommon and were not the primary focus of this work.

Primary closure remains the simplest and often the preferred option for minor cheek defects, provided there is sufficient skin laxity and that the natural facial subunits and relaxed skin tension lines are respected [8,10,40,41]. In our study, primary closure was used for smaller lesions, particularly in regions with good tissue redundancy, such as the upper and lower cheeks and the nasolabial fold. Squamous cell carcinoma was rarely managed with primary closure, which is consistent with its more aggressive biological behavior and the need for broader and deeper resections [41,42]. These observations support current recommendations that primary closure should be reserved for small, low-risk lesions in favorable locations.

Local flaps formed the cornerstone of reconstruction in this study and were used in almost half of all patients. This is consistent with other reports that emphasize the advantages of advancement, rotation, and transposition flaps for cheek reconstruction [30,31,36,37,43]. Local flaps provide an excellent color and texture match, preserve skin thickness, and allow restoration of natural contour lines and aesthetic borders. In our cohort, they were especially useful in the upper cheek, paranasal area, and eyelid region, where primary closure or grafting can lead to eyelid malposition, distortion of the alar base, or conspicuous scarring. Compared with studies that focus on substantial defects and on regional or pedicled perforator flaps [32,33,34,44], our results indicate that local flaps are also well suited to small and medium cheek defects whenever anatomical constraints or aesthetic demands limit the use of simple closure.

Skin grafts were used in a smaller proportion of patients but played a clear role in defined situations. Grafts were selected for older patients, for larger defects, and especially for lesions that involved more than two anatomical regions or extended into the lower eyelid area. These indications are consistent with previous work, in which skin grafts are employed for superficial defects in areas with limited tissue mobility, for example, after prior operations or radiotherapy [11,32,39,43]. Procedures involving grafts were the most time-consuming and resulted in the most extended hospital stays, reflecting the complexity of the underlying defects and the more frequent use of general anesthesia. Despite the well-known disadvantages of grafts, such as color and texture mismatch and the risk of contracture, our experience confirms that they remain a helpful option, particularly in elderly patients with limited reconstructive reserves.

Perioperative outcomes were favorable across all reconstructive methods. The mean operative time and the mean length of hospital stay were comparable with those reported in other series of cheek tumor excision. As expected, procedures, including skin grafts, required more operating time and longer admission, whereas reconstructions with local flaps were shorter despite their technical complexity. The type of reconstruction also influenced the choice of anesthesia. Most primary closures and a large proportion of local flaps were performed under local anesthesia, whereas skin grafts and some flap procedures were carried out under general anesthesia. This pattern is consistent with typical practice in facial skin cancer surgery. It supports the feasibility of managing most cheek reconstructions in an ambulatory or short-stay setting, even in older patients [43,45,46].

Oncologic outcomes further support the safety of the adopted strategies. Margin-negative excision was achieved in the great majority of patients. Local recurrence was uncommon and occurred more often after non-radical excision, which is consistent with earlier work showing that positive or very close margins are associated with a higher risk of relapse [47]. In the margin-negative group, recurrences were rare and mainly basal cell carcinomas, which are known to recur even when histologic margins are clear when the excision width is small. The relatively narrow margins used in our cohort, especially in cases reconstructed with flaps and grafts, therefore appear oncologically acceptable for selected cheek tumors and are in keeping with modern concepts of risk-adapted margins in cosmetically sensitive facial areas [48,49].

The overall complication rate was low. Only two patients developed postoperative complications, and both were treated with local flaps. These complications were minor, could be managed conservatively, and did not cause permanent functional deficits. This rate and pattern of complications are comparable to or better than those reported in other series of cheek and midface reconstruction, in which partial flap necrosis, hematoma, infection, and hypertrophic scarring are typically described [32,34,35,44]. Only a few patients required secondary scar revision, which suggests that aesthetic outcomes were generally satisfactory on clinical examination. Although patient-reported outcome measures were not collected formally, the overall impression at follow-up was that both function and appearance were acceptable.

**Figure 5:**
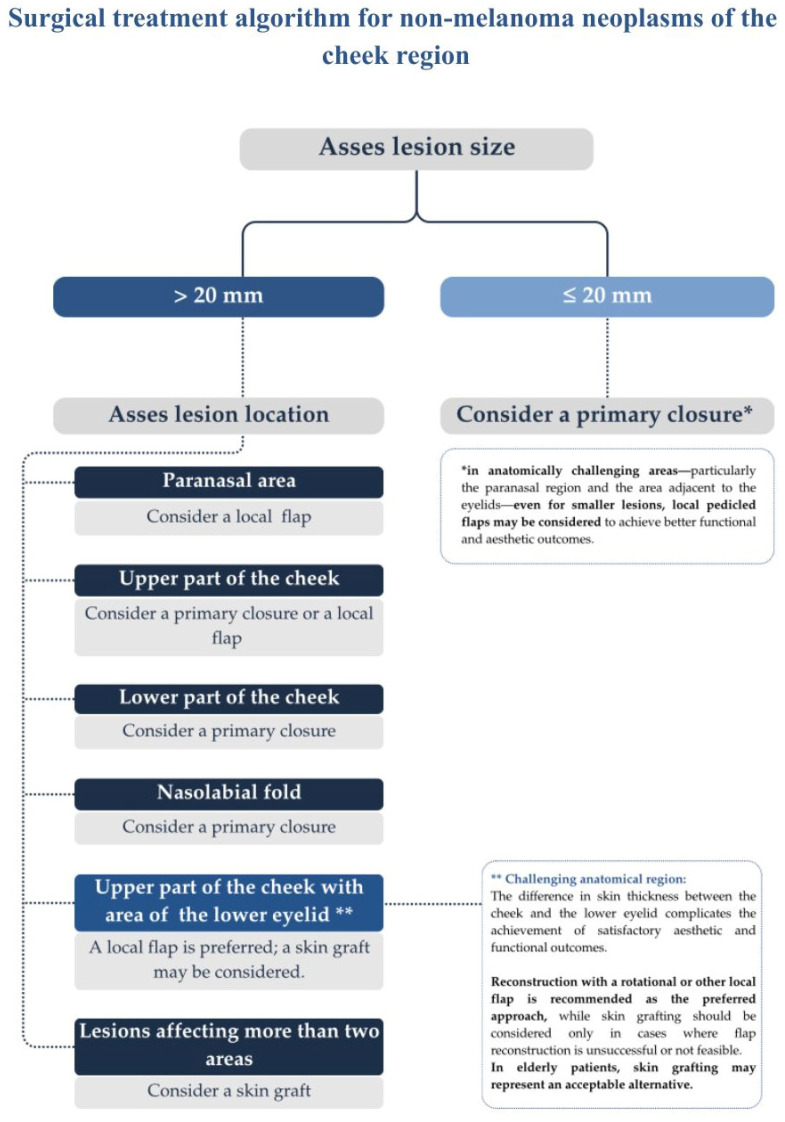
Decision diagram presenting the algorithm for selecting the surgical approach for a cheek skin defect among primary closure, skin graft, or local flap.

A central contribution of this study is the development of a straightforward algorithm that links lesion size and anatomical location to an appropriate reconstructive technique. Statistical analyses showed that lesion diameter and cheek subsite together determined reconstructive complexity. A value of 20 mm emerged as an applicable threshold separating defects usually amenable to primary closure from those that more often require a flap or a graft. The resulting treatment pathway is summarized in the proposed scheme for non-melanoma neoplasms of the cheek region (Figure 5). For lesions of up to twenty mm, primary closure is usually sufficient. However, in anatomically demanding areas such as the paranasal and eyelid regions, local flaps may provide better functional and aesthetic results. For lesions larger than 20 mm, the choice of reconstruction depends mainly on the lesion location. Paranasal defects are best treated with local flaps. Upper cheek lesions can often be closed primarily or reconstructed with a flap. In contrast, lower cheek and nasolabial fold lesions are usually suitable for primary closure due to greater skin redundancy. When the upper cheek defect involves the lower eyelid, local flap reconstruction is recommended as the first option, and skin grafting can be considered when flap reconstruction is not feasible or in elderly patients. Extensive lesions that involve more than two anatomical areas are treated with skin grafting to ensure adequate coverage and avoid excessive tension. The algorithm should be understood as a guide rather than a rigid rule, since reconstructive decisions must always take into account individual patient factors such as age, comorbidities, skin quality, previous surgery or radiotherapy, and patient expectations.

The 20 mm boundary in the proposed workflow should be viewed as a pragmatic, cohort-derived framework rather than a definitive decision threshold. Reconstruction choice in cheek skin defects is multifactorial and depends not only on size but also on subsite, tissue laxity, defect depth, and anticipated closure tension. In this retrospective dataset, we used the 20 mm split to convey a clinically intuitive pattern observed in our cohort: a predominance of primary closure below this size and an increasing need for flaps or grafts above it. Future studies should externally validate this split in independent cohorts and apply multivariable modeling (e.g., logistic regression with adjusted ORs) or data-driven approaches (e.g., CART with cross-validation) to identify robust and generalizable decision rules.

Given the considerable anatomical variability of cheek skin across the population, it is essential to underscore that the proposed algorithm reflects a recommended approach derived from a single-center cohort. In clinical practice, surgical technique selection must be individualized, with careful consideration of patient-specific factors such as skin laxity, tissue quality, age, and comorbidities.

## 5. Study Limitations

This study has several limitations that should be considered when interpreting the results. First, the retrospective design carries the risk of incomplete or inconsistent documentation and does not allow complete control over confounding factors. Second, the analysis was conducted in a single tertiary center, which may limit the generalizability of the findings to other settings with different patient populations or practice patterns. Third, group sizes were unequal, and the number of patients treated with skin grafts was relatively small, thereby reducing statistical power for some comparisons. Fourth, allocation to a given reconstructive method was influenced not only by lesion size and location but also by patient comorbidities, cooperation, and surgeon preference, so selection bias cannot be excluded. Fifth, the follow-up period was limited and focused mainly on early oncologic outcomes and clinical assessment of scars. Standardized patient-reported outcome measures for function and aesthetics were not collected, and the long-term stability of the results could not be fully evaluated. Sixth, lesion size was available as the greatest clinical diameter, whereas the actual post-excision defect size, defect depth, and closure tension were not systematically recorded in this retrospective dataset. Although excision margins were relatively standardized (4 mm for BCC and 6 mm for SCC, i.e., a 2 mm per-side difference), using lesion diameter as a surrogate may have introduced measurement error, particularly in borderline cases near the proposed size threshold. Finally, histopathological high-risk features, such as tumor grading, perineural invasion, and lymphovascular invasion, were not included in the analyses because they did not guide reconstructive decisions and were typically available only from the final postoperative specimen. Standardized patient-reported outcome measures for function and aesthetics were not collected, and the long-term stability of the results could not be fully evaluated. Prospective studies with longer follow-up and with formal assessment of quality of life would help to address these issues.

## 6. Conclusions

Surgical reconstruction of cheek skin defects after excision of non-melanoma skin tumors can be challenging because it must combine oncologic safety with preservation of facial function and appearance. In this single-center cohort, most defects were small or medium and could be managed with primary closure, local flaps, or skin grafts with low complication and recurrence rates.

The present study shows that lesion size and anatomical location are the main factors that determine reconstructive complexity. A threshold of 20 mm and a simple subdivision of the cheek into five regions allowed us to develop an algorithm that guides the choice between primary closure, local flap reconstruction, and skin grafting. This algorithm provides a practical framework for planning reconstruction of cheek defects and may support more consistent decision-making among surgeons.

Future multicenter and prospective studies are needed to validate this approach in other populations, refine the proposed thresholds, and assess long-term functional and aesthetic outcomes, including patients’ perspectives.

## Figures and Tables

**Table 1:** Baseline demographic, clinical, and histopathological characteristics of the study cohort.

Total number of patients (n, %)	129 (100%)
Age, [years], mean (SD)	64.51 (±18.56)
Gender (n, %)	
Female (n, %)	69 (53.49%)
Male (n, %)	60 (46.51%)
BMI [kg/m^2^], mean (SD)	26.54 (±4.70)
Smoking history (n, %)	
Non-smoker	99 (76.74%)
Smoker	30 (23.26%)
Alcohol consumption (n, %)	
Non-drinker	101 (78.30%)
Occasional drinker (less than 3 times per week)	28 (21.70%)
Histopathological diagnosis (n, %)	
Squamous cell carcinoma	22 (17.05%)
Basal cell carcinoma	61 (47.29%)
Benign lesions	46 (35.66%)
• Cutaneous melanocytic nevus (16 cases)	
• Epidermoid cyst (13 cases)	
• Hemangioma (6 cases)	
• Seborrheic keratosis (4 cases)	
• Actinic keratosis (4 cases)	
• Fibroma (2 cases)	
Lesion location (n, %)	
Paranasal area	14 (10.85%)
Upper half of the cheek, excluding the lower eyelid subunit	50 (38.76%)
Upper half of the cheek and the lower eyelid subunit	32 (24.81%)
Lower half of the cheek	10 (7.75%)
Nasolabial fold area	10 (7.75%)
Involving at least two anatomical regions	13 (10.08%)
Lesion site (n, %)	
Right	86 (66.67%)
Left	43 (33.33%)
Lesion measurement [mm], mean (SD)	19.75 (±12.93)
Surgical technique (n, %)	
primary closure	53 (41.09%)
skin graft	14 (10.85%)
local flap	62 (48.06%)

**Table 2:** Characteristics of surgical procedures and early oncologic outcomes.

Duration of the surgical procedure [min], mean (SD)	63.32 (±36.05)
Time of hospitalization [days], mean (SD)	2.59 (±3.56)
Resection type (n, %)	
• R0	123 (95.35%)
• R1	6 (4.65%)
Type of anesthesia (n, %)	
• Local anesthesia	96 (74.42%)
• General anesthesia	33 (25.58%)
Complications occurrence (n, %)	2 (1.56%) in the local flaps group:
	wound dehiscence with hematoma
	wound dehiscence with suppuration
Local recurrences (n, %)	
Total	5 (3.91%)
In non-radical excision (R1) group (n, %)	1 out of 6 R1 patients (16.67%)
• One SCC patient-flap group	
• Minimal excision margin [mm]	3
In radical excision (R0) group (n, %)	4 out of 123 R0 patients (3.25%)
• 3 BCC patients:	
Minimal excision margin [mm], mean (SD)	1.15 (±0.96)
• flap group	2.25
• graft group	0.7
• primary closure group	0.5
Time to onset of local recurrence, [months], mean (SD)	12.83 (±4.75)

**Table 3:** Demographic and clinical characteristics of patients according to surgical technique.

Surgical Technique	Primary Closure	Skin Grafts	Local Flaps	*p* Value
Age, [years], mean (SD)	61.56 (±17.97)	78.69 (±12.73)	63.63 (±18.85)	0.005
Gender, female (n, %)	24 (45.28%)	9 (64.29%)	36 (58.06%)	0.271
BMI [kg/m^2^], mean (SD)	26.43 (±5.24)	25.98 (±4.04)	26.75 (±4.34)	0.813
Smoking history				0.491
• Non-smoker	38 (71.70%)	12 (85.71%)	48 (77.42%)	
• Smoker	15 (28.30%)	2 (14.29%)	14 (22.58%)	
Alcohol consumption (n, %)				0.918
• Non-drinker	39 (73.58%)	11 (78.57%)	51 (82.26%)	
• Occasional drinker (<3 times per week)	14 (26.42%)	3 (21.43%)	11 (17.74%)	
Histopathological diagnosis (n, %)				<0.001
• SCC	0 (0.00%)	6 (42.86%)	16 (25.81%)	
• BCC	28 (52.83%)	8 (57.14%)	26 (41.94%)	
• Benign tumor	25 (47.17%)	0 (0.00%)	20 (32.26%)	
Lesion location (n, %)				<0.001
• Paranasal area	4 (7.55%)	0 (0.00%)	9 (14.52%)	
• Upper half of the cheek	23 (43.40%)	1 (7.14%)	26 (41.94%)	
• Upper half of the cheek and the area of the lower eyelid	8 (15.09%)	5 (35.71%)	18 (29.03%)	
• Lower half of the cheek	10 (18.87%)	0 (0.00%)	1 (1.61%)	
• Nasolabial fold area	6 (11.32%)	0 (0.00%)	4 (6.45%)	
• Involving at least two anatomical regions	2 (3.77%)	8 (57.14%)	4 (6.45%)	
Lesion measurement [mm], mean (SD)	15.72 (±9.22)	26.00 (±19.38)	21.98 (±13.21)	0.03

**Table 4:** Perioperative and early oncologic outcomes according to surgical technique.

Procedure Type	Primary Closure	Skin Grafts	Local Flaps	*p* Value
Duration of the surgical procedure [min], mean (SD)	66.37 (±38.62)	88.89 (±51.28)	56.88 (±29.22)	<0.001
Time of hospitalization [days], mean (SD)	1.23 (±0.78)	5.62 (±2.63)	3.18 (±4.66)	<0.001
Resection type (n, %)				0.03
• R0	53 (100.00%)	14 (100%)	56 (90.32%)	
• R1	0 (0.00%)	0 (0.00%)	6 (9.84%)	
Type of anesthesia (n, %)				<0.001
• Local anesthesia	50 (94.34%)	5 (35.71%)	41 (66.13%)	
• General anesthesia	3 (5.66%)	9 (64.29%)	21 (33.87%)	
Local recurrences (n, %)	1 (1.89%)	1 (7.14%)	3 (4.84%)	0.169
Time to onset of local recurrence, [months], mean (SD)	12	12	15 (±6.08)	0.616
Secondary corrective procedures (n, %)	scar modeling in 2 patients (3.77%)	no scar remodeling	3 (4.84%)	0.652
Survival over the follow-up period (n, %)	53 (100%)	13 (92.86%)	56 (90.32%)	0.05
Death (n, %)		1 (7.14%)	6 (9.68%)	

**Table 5:** Mean lesion size and intergroup differences across surgical techniques.

Surgical Technique	Number of Patients	Mean Defect Size [mm]	Standard Deviation	Significant Differences (Games-Howell)
Primary closure	53	15.72	±9.22	Smaller than skin graft and local flap groups (*p* = 0.018)
Skin graft	14	26.00	±19.38	Greater than primary closure group (*p* = 0.018)
Flap reconstruction	62	21.98	±13.21	Greater than primary closure group (*p* = 0.018)

## Data Availability

The datasets presented in this article are not readily available because the data are part of an ongoing study. Requests to access the datasets should be directed to J.B.

## Abbreviations

The following abbreviations are used in this manuscript:

SCC	Squamous cell carcinoma
BCC	Basal cell carcinoma
NMSC	Non-melanoma skin cancer
RSTL	Relaxed skin tension lines
